# Removal of Antimony in Wastewater by Antimony Tolerant Sulfate-Reducing Bacteria Isolated from Municipal Sludge

**DOI:** 10.3390/ijms23031594

**Published:** 2022-01-29

**Authors:** He Li, Yue Fei, Shuwen Xue, Gege Zhang, Ziqi Bian, Fanfan Guo, Li Wang, Ruiqing Chai, Shuqi Zhang, Zhenyu Cui, Shiwei Wang, Jun Zhang

**Affiliations:** 1 College of Life Sciences, Northwest University, Xi’an 710069, China; lihe@stumail.nwu.edu.cn (H.L.); m18291802944@163.com (Y.F.); xuesw@nwu.edu.cn (S.X.); 202032708@stumail.nwu.edu.cn (G.Z.); bianziqi@stumail.nwn.edu.cn (Z.B.); guofanfan@stumail.nwu.edu.cnm (F.G.); wangli@stumail.nwu.edu.cn (L.W.); 2019113110@stumail.nwu.edu.cn (R.C.); 2018113050@stumail.nwu.edu.cn (S.Z.); cuizhenyu@stumail.nwu.edu.cn (Z.C.); 2 Shaanxi Key Laboratory of Animal Conservation, College of Life Sciences, Northwest University, Xi’an 710069, China; 3 Key Laboratory of Resources Biology and Biotechnology in Western China, Ministry of Education, Northwest University, Xi’an 710069, China

**Keywords:** antimony tolerant, sulfate-reducing bacterial strains, antimony removal

## Abstract

Antimony (Sb), a global and priority controlled pollutant, causes severe environmental issues. Bioremediation by microbial communities containing sulfate-reducing bacteria (SRB) is considered to be among the safest, economical, and environmentally friendly methods to remove Sb from wastewater. However, the roles of SRB species in these communities remain uncertain, and pure cultures of bacteria that may be highly efficient have not yet been developed for Sb removal. In this study, an Sb tolerant community was enriched from municipal sludge, and molecular ecological analysis showed that *Escherichia* (40%) and *Desulfovibrio* (15%) were the dominant bacteria. Further isolation and identification showed that the enriched SRB strains were closely related to *Cupidesulfovibrio oxamicus*, based on the molecular analyses of 16S rRNA and *dsrB* genes. Among them, a strain named SRB49 exhibited the highest activity in removal of Sb(V). SRB49 was able to remove 95% of Sb(V) at a concentration of 100 mg/L within 48 h under optimum conditions: a temperature of 37–40 °C, an initial pH value of 8, 4 mM of sulfate, and an initial redox potential of 145–229 mV. SEM-EDX analysis showed that SRB49 did not adsorb Sb(V) but reduced and precipitated Sb(V) via the formation of Sb_2_S_3_. The results demonstrated the potential roles that pure cultures of SRB species may play in Sb removal and the use of Sb-tolerant SRB strains for Sb remediation.

## 1. Introduction

Antimony (Sb) is a metalloid widely used in various industrial fields, such as in the production of flame retardants, batteries, plastics, and catalysts [1]. With rapidly increasing use, Sb is currently the ninth most mined metal in the world [2]. Due to natural processes and human activities such as mining and smelting, coal combustion, waste incineration, and spent ammunition, severe Sb pollution is emerging worldwide, especially in China, which has the richest antimony ore resources and the highest mining activity [2,3]. Wastewaters from mines and smelting factories containing high levels of Sb(V) are major pollution sources that threaten the ecosystems of the receiving waters as well as human health through drinking water and the food chain [4]. Due to its toxicity, being a suspected carcinogen, and its adverse effects on ecosystems [5], Sb and its compounds are considered as priority pollutants by the United States Environmental Protection Agency and the European Union [1]. Therefore, treatments for Sb pollution have become a research focus worldwide over the last decade [1,4,6].

As compared to various physical and chemical methods, such as coagulation, electrodeposition, adsorption, and ionic exchange, bioremediation methods are more cost effective with less process complexity and secondary pollution [7]. Through biosorption, oxidation, reduction, and/or methylation, microorganisms play an important role in the mitigation of Sb contamination. Metal sulfide precipitation by sulfate-reducing bacteria (SRB) has potential as a method for removing Sb from water [8]. Sb mainly has two oxidation states in an aqueous environment, with Sb(V) and Sb(III) as the predominant species in oxic and anoxic settings, respectively [8]. Under anaerobic conditions, H_2_S produced through the sulfate reduction of SRB can reduce Sb(V) to Sb(III), coupled with the subsequent formation of Sb(III) precipitate Sb_2_S_3_ (stibnite), which decreases Sb mobility and its threat to drinking water [9,10].

Several studies have been reported in recent years regarding bioreduction by SRB flora in various anaerobic environments [7,8,11,12]. The bacterial communities in SRB enriched cultures were reported to remove 83.5–90% of Sb(V) at 2.5–50 mg/L but only 48.1% at 75 mg/L [13]. The presence of 100–200 mg/L Fe(II) [7] or 4 mM sulfate [8] enhanced the Sb removal efficiency of SRB flora. Although the Sb concentration in the sediment of rivers polluted by antimony ore can be as high as 17,000 mg/kg [14], there has been a paucity of reports detailing the treatment of high Sb concentrations in wastewater, which may be due to the toxicity of Sb to bacteria [15].

The aforementioned studies were based on the activity of SRB enriched cultures with complex bacterial community structures. Although SRB species were originally thought to be the major functional group responsible for the reduction and precipitation of Sb(V), the roles of the SRB species in these communities have remained uncertain as a highly efficient pure culture of SRB had yet to be developed for Sb removal. However, when a pure culture strain of SRB, *Desulfovibrio vulgaris* DP4, was reported to have no Sb(V) removal ability when incubated with 2 mM Sb(V), that understanding could not be supported [12]. Moreover, instead of precipitating Sb and reducing the mobility of Sb, *Desulfovibrio vulgaris* DP4 was reported to increase the mobility of Sb(V) adsorbed by goethite combined with generating thioantimonate [16]. SRB species such as *Desulfovibrio* spp. and *Desulfomicrobium* spp. have been found in surprisingly low proportions (<1%) in the bacterial community of SRB enriched cultures and in an Sb polluted environment [7,8,12]. These results suggested that the roles of these SRB species in antimony mobility need to be further investigated. In addition, environmental factors, including initial pH, temperature, redox potential, sulfate concentration, co-contamination of heavy metal ions, and the existence of Fe^0^, influence the treatment efficiency of Sb contaminated wastewaters by SRB species, but they have rarely studied or analyzed.

To the best of our knowledge, no study concerning the precipitation and the removal of Sb(V) from wastewater has been conducted based on the activity of a pure culture of SRB, and no strains of antimony resistant SRB have been reported. In addition, the environmental parameters influencing the Sb(V) treatment efficiency of SRB have not been examined in a pure culture system. In this study, we enriched and isolated Sb resistant SRB strains from a municipal sewerage, refined the treatment parameters to remove Sb(V) from wastewater, and analyzed the mechanisms of Sb(V) removal.

## 2. Results

### 2.1. Enrichment of SRB Community with Sb(V) Removal Activity and Its Community Analysis

**Figure 1:**
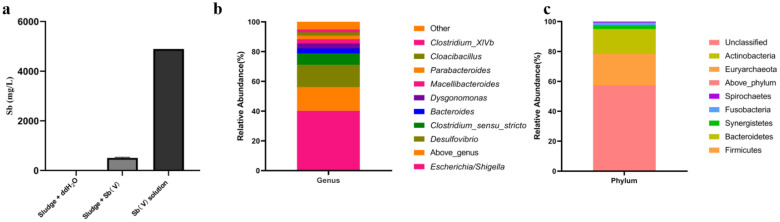
The removal of Sb in municipal sludge after 14-day incubation (**a**) and the community composition of SRB enriched community at the genus (**b**) and phylum levels (**c**).

To obtain a highly Sb tolerant bacterial community, a 14-day incubation of municipal sludge with a high concentration of Sb(V) was performed. The results showed that the municipal sludge reduced 90% of Sb(V), while no reduction of total Sb was observed in the control group with only Sb(V) and ddH_2_O (Figure 1a), indicating that the Sb(V) was stable. After SRB enrichment, the Sb removal efficiency of the enriched community increased to 96% within 48 h. The illumina sequencing of the 16 S rRNA gene of the SRB enriched community obtained a total of 48,303 sequences and identified 219 operational taxonomic units (OTUs) at a 97% sequence similarity cutoff. The Simpson and Shannon diversity indices were 0.32 and 2.95, respectively. The relative abundance at the genus and phylum levels are shown in Figure 1b,c. The bacterial community at the level of phylum was mainly composed of Proteobacteria (57.3%), Firmicutes (21.1%), and Bacteroidetes (16.6%). More than 84% of the OTUs were identified at the genus level, and the most dominant genera in the SRB enriched culture were *Escherichia* (40.2%) followed by *Desulfovibrio* (15.0%). The less abundant genera included *Clostridium sensu stricto* (7.6%), *Bacteroides* (3.4%), *Dysgonomonas* (3.3%), *Macellibacteroides* (2.9%), *Parabacteroides* (2.3%), *Cloacibacillus* (2.2%), and *Clostridium_XlVb* (1.9%) (Figure 1c).

### 2.2. Isolation and Identification of Sb Tolerant SRB Strains

**Figure 2:**
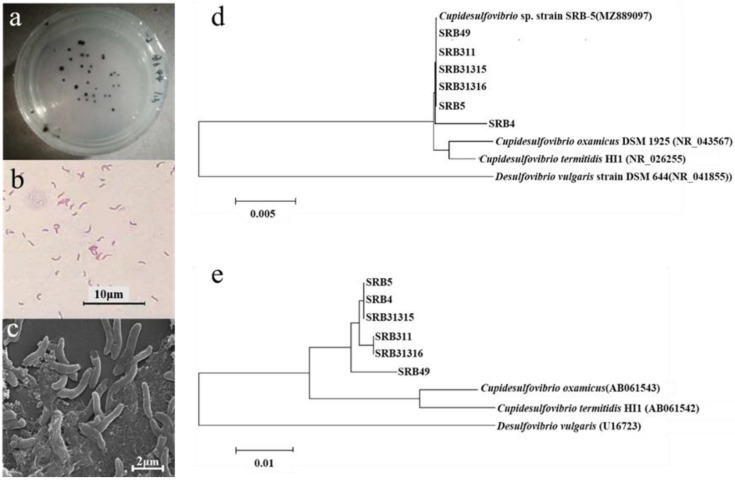
Morphology of SRB isolates on the plate (**a**), under optical microscope (**b**), and scanning electron microscopes (**c**). Phylogenetic tree of all SRB strains based on 1.4 Kb of 16S rRNA gene (**d**) and 300 bp of *dsrB* gene (**e**) sequence analysis.

Four strains were isolated and named SRB311, SRB31315, SRB31316, and SRB49. All the strains were Gram negative, non spore forming, vibrio shaped bacteria with a size of 3 ± 0.8 μm × 0.4 ± 0.03 μm (Figure 2a–c).

Based on the analysis of 1.4 kb of the 16S rRNA gene sequence, all the strains shared a 100% sequence similarity to each other and were closely related to the *Cupidesulfovibrio* strains, a genus recently separated from genus *Desulfovibrio*. The most closely related strains were *C. oxamicus* and *C. termitidis,* with sequence similarities of 99.5% and 98.3%, respectively (Figure 2d). To further distinguish the strains, the sequences of the *dsrB* gene were analyzed and all strains were classified to the three clusters (Figure 2e) closest related to *C. oxamicus* and *C. termitidis*. SRB49 was closest related to *C. oxamicus* and *C. termitidis*, with a sequence similarity of 97.4% and 97.1%, respectively. SRB311 and SRB31316 had the same *dsrB* sequences and were the closest related to *C. oxamicus* (97.7% similarity) and *C. termitidis* (97.4% similarity) (Figure 2e). Therefore, the four strains were identified as *C. oxamicus*.

### 2.3. Sb(V) Removal by Pure Culture of SRB Strains

**Figure 3:**
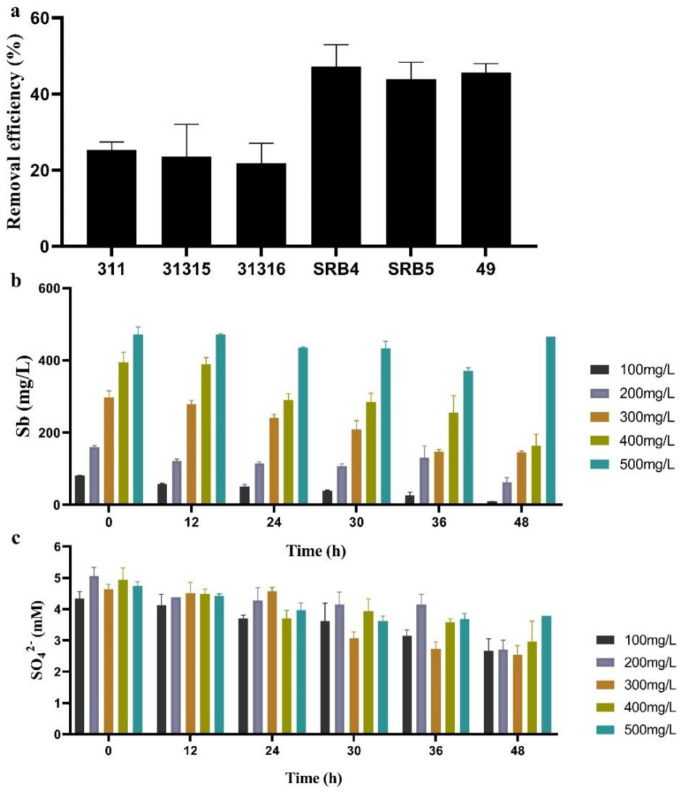
The Sb-removal efficiency of all SRB strains within 24 h (**a**), the performance of SRB49 on Sb removal (**b**) and sulfate consumption (**c**) within 48 h in different Sb(V) concentrations.

All SRB strains exhibited the ability to remove Sb from synthetic wastewater, and SRB4, SRB5, and SRB49 (44–47.2%) had significantly higher Sb-removal efficiency, as compared to other strains (22–29.3%) within 24 h (Figure 3a). SRB49 showed the highest Sb(V)-removal efficiency and thus was selected for the following experiments.

For the analysis of Sb(V) tolerance and removal efficiency by SRB49 at higher Sb concentrations, SRB49 was incubated in synthetic wastewater with Sb(V) concentrations of 100–500 mg/L for 48 h (Figure 3b). SRB49 exhibited a high activity of 90.8% removal efficiency against 100 mg/L of Sb(V). At higher Sb(V) concentrations of 200, 300, and 400 mg/L, the final removal of Sb was reduced to 60%, 51%, and 58%, respectively, which indicated that the activity of SRB49 in this treatment was partially inhibited (Figure 3b). The sulfate consumptions were not significantly different among treatments in Sb(V) concentrations of 100–400 mg/L (Figure 3c), suggesting that the growth of SRB49 was not affected by Sb(V) concentrations of up to 400 mg/L. At an Sb concentration of 500 mg/L, SRB49 showed no sulfate consumption within 48 h. This result indicated that the growth and Sb(V) removal ability of SRB49 was thoroughly inhibited by 500 mg/L of Sb(V).

### 2.4. Effects of Different Culture Parameters on Sb(V) Removal by SRB49

#### 2.4.1. Effect of Redox Potential on the Removal of Sb(V)

**Figure 4:**
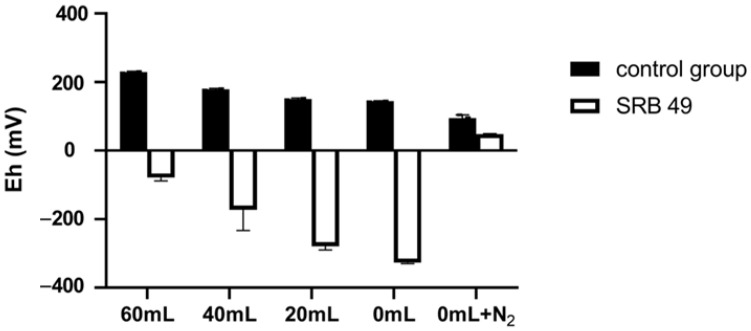
The redox potential in the synthetic wastewater with different upper air after incubated with and without SRB49 for 48 h.

**Figure 5:**
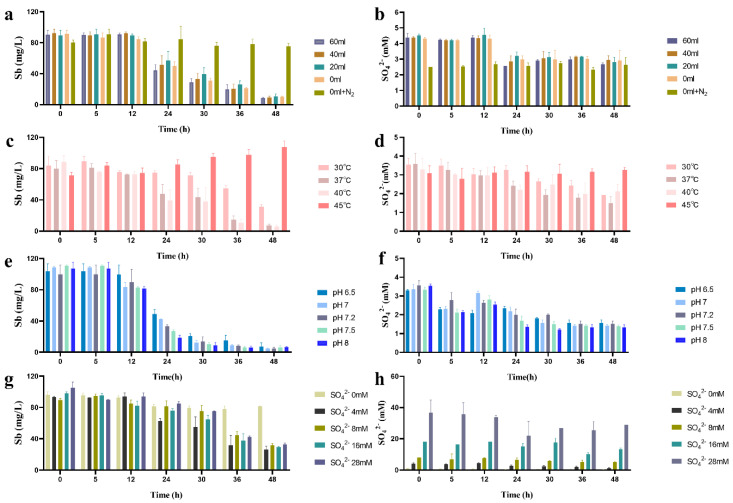
Sb-removal (**a**,**c**,**e**,**g**) and sulfate consumption (**b**,**d**,**f**,**h**) by SRB49 under different culture parameters.

The redox potential in the synthetic wastewater without bacteria decreased from 229 to 145 mV with the decreased volume of upper air, and treatment with 0 mL upper air plus an N_2_ flush and deoxidizer further reduced the initial Eh to 95 mV (Figure 4). After incubation with SRB49 for 48 h, the Eh value turned negative and sharply decreased from −78.3 to −327 mV with the volume decrease in the upper air (Figure 4). This supported a previous report that had suggested that the growth in SRB had resulted in a strong reduction in the culture [13]. No significant differences were observed in Sb(V) removal efficiency (89.9–90.2%) or sulfate consumption (2 mM) among treatments with different upper air (Figure 5a,b). On the contrary, in the treatment using 0 mL upper air plus N_2_ flush and deoxidizer, neither Sb(V) removal nor sulfate consumption was observed (Figure 5a,b). The Eh value was still positive, with only a slightly decrease. This result indicated that the initial Eh of 229–145 mV was suitable for SRB49 growth and Eh < 95 mV thoroughly inhibited the growth of SRB49. Therefore, 0 mL upper air (initial Eh of 145 mV) were used for the following experiments.

#### 2.4.2. Effect of Temperature, Initial pH, and Sulfate Concentration on the Removal of Sb(V)

The effect of temperature, initial pH, and sulfate concentration on the batch treatment of Sb(V) by SRB49 was examined within a temperature range of 30–45 °C, pH values of 6.5–8, and sulfate concentrations of 2–28 mM, respectively. After a 48-h incubation, more than 90% of the aqueous Sb was removed between the temperature range of 37–40 °C (Figure 5c), while only 59.4% of the aqueous Sb was removed at 30 °C. The sulfate consumption at 30 °C was 0 mM within the first 24 h and then reached the same value as treatments at 37 °C and 40 °C within 48 h (Figure 5d), which indicated the reduction in Sb-removal efficiency was due to the delayed growth in SRB49 at temperatures ≤30 °C. No removal of Sb and sulfate consumption was observed in the treatment at 45 °C, indicating that the growth of SRB49 strain was thoroughly inhibited.

After a 48-h incubation, over 93–96% of the aqueous Sb was removed at the initial pH range of 6.5–8, and no significant differences were found among all the treatments, which indicated that SRB49 could adapt to a wide range of pH values. The Sb concentration only varied significantly among treatments within a 24-h incubation (Figure 5e). The Sb removal efficiency within 24 h increased from 52–82% with the increase in the initial pH of 6.5 to 8, wherein a high removal rate of 3.69 mg/L·h was reached. The sulfate consumption ranged from 1.74 to 2.21 mM (Figure 5f). Taken together, these results showed that SRB49 could exhibit high Sb-removal activity across a wide range of pH values and the different initial pH values affected the rate of antimony removal.

After a 48-h incubation, 89.8–90.3% of the aqueous Sb was removed over an initial sulfate concentration of 4–28 mM (Figure 5g). No significant difference in Sb removal and sulfate consumption were found among all the treatments, which indicated that SRB49 could adapt to high sulfate concentrations (Figure 5h). The treatment without sulfate showed no Sb removal (Figure 5g,h). Within the first 24 h, the Sb removal was 50.9% in a treatment with 4 mM of sulfate, which was significantly higher than that of other groups (18–20%), suggesting that the Sb removal was faster at 4 mM sulfate within the first 24 h.

### 2.5. SEM-EDX Characterization Analysis

**Figure 6:**
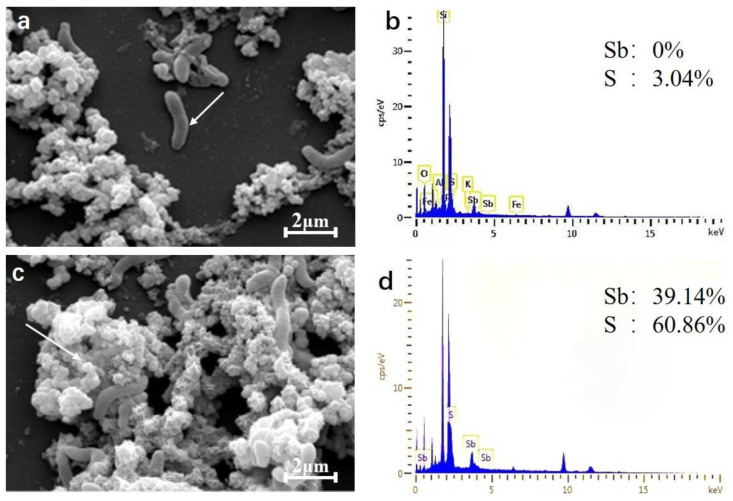
Scanning electron micrographs of cells (**a**) and mineral precipitates (**c**) and EDX spectrum on the cell surface (**b**) and mineral precipitates (**d**) shown by white arrows.

The precipitates after incubation with SRB49 for 48 h were characterized by scanning electron microscopy-energy dispersive X-ray (SEM-EDX) (Figure 6). The SRB bacteria were entangled with amorphous precipitates that were typically less than 1 µm in diameter (Figure 6a,c). The energy dispersive X-ray (EDX) analysis on the surface of the bacteria showed that there was no Sb element on the bacterial surface (Figure 6a,b), suggesting that the bacteria did not absorb any Sb. EDX analysis of the precipitates of SRB49 detected the Sb and S peaks and found that the ratio of Sb/S was approximately 2:3 (Figure 6c,d), which indicated the formation of Sb_2_S_3_.

## 3. Discussion

### 3.1. Community Analysis of SRB Community Enrichment Culture

After enrichment, the SRB community was dominated by a phylum of Proteobacteria (57.3%), Firmicutes (21.1%), and Bacteroidetes (16.6%). They were ubiquitously found in various anaerobic sludge and SRB enriched cultures for Sb(V) bioreduction [7,8]. These results were in accordance with previous reports that showed that Proteobacteria was overwhelmingly dominant [8]. The most abundant genus, *Escherichia*, was composed of only one OTU, which accounted for 40.2% of the total. *E. coli* was also found as the most dominant specie in lactate fed SRB enriched cultures with Sb removal activity [7]. Its low nutrition requirements and wide distribution in various anaerobic environments make it difficult to remove when purifying and culturing SRB [7]. Genus *Desulfovibrio* was the second dominant genus (15.0%) in the SRB enriched community and also the only SRB genus found in this study. The proportion of SRB was much higher in this study, as compared to that found in other reports (e.g., 0.55–1%), which suggested that enrichment by incubation with high levels of Sb(V) probably promoted the growth in Sb tolerant *Desulfovibrio* species and the possibility of sulfate bioreduction. *Pseudomonas* and *Geobacter* have been identified as key genera for Sb(V) bioreduction [7,8] and were not detected in this study. Therefore, the SRB enriched culture showed significant differences in community composition as well as a high Sb(V) removal activity (96%), which indicated that diverse bacteria were involved in Sb(V) bioreduction.

### 3.2. Sb Removal by Sb Tolerant SRB Strains

In this study, the SRB enriched culture and the purified SRB strains were tested for precipitates and Sb(V) removal activity from wastewater with >90% removal efficiency in an Sb(V) concentration of 100 mg/L within 48 h. When the Sb(V) concentration was increased to 400 mg/L, SRB49 still exhibited 58% removal efficiency. In previous experiments, SRB enriched cultures that showed high Sb removal efficiency were generally conducted in an Sb(V) concentration of less than 25 mg/L [7,9,17]. Zhang et al. reported a removal efficiency of 83.5–90% at an Sb(V) concentration of 2.5–50 mg/L but only 48.1% at 75 mg/L of Sb(V) [13]. Moreover, SRB49 could rapidly reduce 82% of Sb(V) within 24 h and reach over 94.2% removal within 36 h, with the highest removal rates, of 3.69 and 2.81 mg/L·h, respectively. The Sb removal efficiency of SRB enriched culture was generally reported to reach the highest values after 3–9 days of incubation [7,9,13,17]. The reduction rates for pure antimonate-reducing bacteria were in the range of 0.33–2.88 mg/L·h [18,19]. Therefore, the Sb tolerant strain, SRB49, used in this study exhibited a high rate of Sb removal in high Sb(V) concentrations within a short time. The precipitate was Sb_2_S_3_ and had no adsorption of Sb on the surface of the SRB49, which indicated the SRB49 mainly removed Sb(V) through metal sulfide precipitation. Pure strains of SRB, *D. vulgaris* DP4, was reported to exhibit no Sb(V) removal ability when incubated with 2 mM Sb(V) [12] and increased the mobility of Sb(V) adsorbed by goethite [16]. This conflict in the role of SRB species in Sb removal may be explained by the high tolerance of Sb(V) towards SRB49, which could still grow in an Sb(V) concentration in excess of 2 mM (equal to 243.5 mg/L). To the best of our knowledge, this is the first report of Sb tolerant SRB strains with a high Sb(V) removal rate.

### 3.3. Influence of Different Culture Parameters on Sb(V) Removal of SRB49

SRB49 exhibited more than 90% Sb(V) removal efficiency within a broad range of pH values, 6.5–8, temperatures of 37–40 °C, sulfate concentrations of 2–28 mM, and Eh of 145–229 mV. The highest removal efficiency was 95.7% when the pH was 7 at 40 °C and in 4 mM of sulfate. This wide adaption range of SRB49 suggested its potential application for Sb containment control in various environments.

SRB49 showed better growth and Sb removal under an initial Eh of 145–229 mV and no growth under an initial Eh of less than 95 mV, which indicated that the SRB49 isolated in this study was not an obligate anaerobe. SRB has been shown to be active under oxygen ranging from anaerobic to aerobic [20]. The facultative anaerobic SRB of genera *Desulfotomaculum* and *Vibrio* has been isolated from both crude oil and marine corrosive steel and exhibited corrosive functions [21,22]. Mogensen et al. [23] isolated an oxygen-resistant sulfate-reducing strain of DVO5 (T) from activated sludge that could survive under 100% air oxygenation for 120 h. Since Sb has been present mainly as Sb(V) under oxic conditions and remained the predominant species over a wide redox range [13,24], the discovery of facultative SRB capable of Sb bioprecipitation would be beneficial for Sb(V) removal under wider redox ranges and helpful for understanding the biochemical roles of SRB on Sb mobility.

## 4. Materials and Methods

### 4.1. Enrichment of Sb Tolerant Bacterial Community

Municipal sludge was obtained from Ruicheng Sewage Treatment Plant in Yuncheng City, Shanxi Province, China. We inoculated 6 g of sludge into 100 mL of potassium pyroantimonate solution with an Sb(V) concentration of 5 g/L and incubated in 100 mL sealed anaerobic bottles at 37 °C for 14 days. After incubation, 10 mL of sludge was then inoculated in the modified Postgate B medium [9] and incubated at 37 °C for SRB enrichment with an Sb(V) concentration of 100 mg/L [7]. The SRB enriched cultures were then subcultured three times and used for isolation of SRB strains.

### 4.2. Bacterial Community Analysis of SRB Enriched Culture by Illumina Sequencing of 16S rRNA Genes

The bacterial community structure of the SRB enriched culture was analyzed using high throughput sequencing of 16S rDNA. The total DNA of the SRB enriched cultures were extracted using a E.Z.N.A.^®^ Soil DNA Kit (Omega Bio-tek, Norcross, GA, USA), according to the manufacturer’s protocols. Polymerase chain reaction(PCR) amplification of the V4–V5 region of the bacterial 16S rRNA gene was performed on GeneAmp 9700 PCR System (Applied Biosystems, Foster City, CA, USA) using primers 515F–907R. Sequencing and data analysis were carried out by Luojie Company (Luojie, Jinan, China).

### 4.3. Isolation and Molecular Identification of Sb Tolerant SRB Strains

The isolation of pure SRB strains by overlayer plate was conducted as reported previously [25] using the same medium with Fe (II) but no Sb(V) for enrichment. The black colonies on the plates were selected and sub cultured into fresh medium. This procedure was repeated three times until pure isolates were obtained. The purified SRB strains were then preserved at −80 °C. At the end of the experiment, four pure cultures were obtained and analyzed.

The genomic DNA of all the SRB isolates and two SRB strains kindly provided by the Environmental Microbiology Experimental Group of Northwest University were extracted using TIANamp Bacteria DNA kits (Tiangen Biotech, Beijing, China) and following the manufacturer’s instructions. The genomic DNA were examined using 0.7% agarose gel electrophoresis and used as templates for the amplification of 16S rRNA gene using bacterial universal primers 27F and 1492r. The SRB specific functional gene *dsrB* was also amplified using a primer pair of 2060F [26] and DSR4R [27]. The PCR settings for the two genes were used as reported previously [26]. The PCR products were examined by gel electrophoresis and sequenced by Sangon Biotech (Shanghai, China). The SRB isolate sequences were quality trimmed, and compared to reference sequences deposited in the GenBank database using the BLASTN algorithm (http://www.ncbi.nlm.nih.gov/BLAST/, accessed on 16 December 2021), and aligned using BioEdit (version 7.0.5, Manchester, UK). The phylogenetic trees were constructed using MEGA7 software (Mega Limited, Auckland, New Zealand) and the neighbor-joining approach, and the statistical support of hypotheses was assessed using 1000 bootstrap replicates [28].

### 4.4. Sb(V) Removal by SRB Enrichment and Pure Culture of SRB from Synthetic Wastewater

The synthetic wastewater was prepared by adding the necessary amount (g) of potassium pyroantimonate solution to the modified Postgate B medium to reach a final Sb(V) concentration of 100 mg/L. The composition of the medium was consistent with that used for enrichment except that ferrous sulfate was excluded. For the analysis of Sb removal activity of SRB enriched culture, the fresh culture was inoculated by 10% and added to synthetic wastewater and incubated in a sealed saline bottle at 37 °C for 48 h. For the analysis of Sb removal activity of the purified SRB strains, SRB strains of 3 × 10^8^ CFU/mL were inoculated into synthetic wastewater and incubated as described above. Samples were taken by syringe with a long needle at 0 h and 24 h for chemical analysis. To examine the Sb tolerance of SRB strains under higher Sb concentrations, the SRB strain 49 (named as SRB49) with the highest Sb removal efficiency among all six SRB strains was inoculated into synthetic wastewater with different initial Sb(V) concentrations of 100, 200, 300, 400, and 500 mg/L and were incubated at 37 °C for 48 h. Samples were taken at 0, 5, 12, 24, 30, 36, and 48 h. All the batch experiments were conducted in triplicate. The data were presented as the mean values and standard deviations.

### 4.5. Effects of Different Culture Parameters on Sb(V) Removal by SRB Strain

Batch experiments for Sb(V) removal under different culture parameters were conducted using the same procedure as described above. The batch experiments were performed in triplicate in 500 mL glass bottles sealed with rubber stoppers in an Sb(V) concentration of 100 mg/L and incubated at 37 °C for 48 h. At incubation times of 0, 5, 12, 24, 30, 36 and 48 h, 10 mL of the culture aliquots were collected for chemical analysis.

To analyze effects of different initial redox potential on Sb(V) removal, different volumes of synthetic wastewater with an Sb(V) concentration of 100 mg/L was added to 500 mL glass bottles with an upper space of 0, 20, 40, and 60 mL. Another bottle filled with synthetic wastewater without upper space was further flushed with N_2_ for 15 min before autoclave and added 0.5 mg/L filter sterilized ascorbic acid before inoculation.

For effect analysis of temperature on Sb(V) removal, SRB49 strains were cultured at temperatures of 30, 37, 40, and 45 °C. For effect analysis of initial pH and sulfate concentration on Sb(V) removal, SRB49 strains were cultured at initial pH values of 6.5, 7, 7.2, 7.5, and 8; and sulfate concentrations of 0, 4, 8, 16, and 28 mM, respectively.

### 4.6. Chemical Analysis and Statistical Analysis

Sb concentration was determined by inductively coupled mass spectrometry (ICP-MS) using an Agilent 7900 ICP-MS (Tokyo, Japan). The liquid samples were taken from bottles using sterile syringes with long needles and filtered through the 0.22-μm hydrophilic polyethersulfone (PES) membrane. The filtered samples were acidified with HNO_3_, diluted to ppb levels, and filtered again for ICP-MS analysis [29]. The concentration of sulfate was measured by barium chromate spectrophotometry [30]. The pH values and the redox potential were determined by pH meter (Sartorius PB-10, Gottingen, Germany) with and the REDOX potential meter (Leigi, PHS-3C, Shanghai, China). Statistical analysis to determine the significant difference (*p* < 0.05) was performed using GraphPad Prism software (version 6.0, San Diego, CA, USA) using a one way analysis of variance (ANOVA).

### 4.7. SEM-EDX Analysis

The precipitates formed after 48 h treatment of synthetic wastewater by SRB49 were analyzed by SEM-EDX [31]. The precipitates were washed twice with sterile phosphate-buffered saline (PBS), fixed with 2.5% glutaraldehyde, washed with PBS 3 times, dehydrated with ethanol at different gradients, and dried at room temperature for 2 days. Samples were sprayed with gold at 20 kV, 128 μA current, and characterized by scanning electron microscopy (SEM, Hitachis-3000N, Tokyo, Japan) operated at 15 kV with a working distance of 10 mm. For EDX (X-MAXN50) analysis, an accelerating voltage of 20 kV was used to obtain sufficient X-ray counts.

## 5. Conclusions

In this study, high concentrations of Sb in simulated wastewater were removed by a pure culture of an Sb tolerant SRB strain that had been isolated from municipal sludge. Strain SRB49 was able to remove 95.7% of Sb(V) at a concentration of 100 mg/L within 48 h under optimum conditions: a temperature of 37–40 °C, an initial pH value of 8, 4 mM of sulfate, and an initial redox potential of 145–229 mV. This strain was identified as *C. oxamicus*. The mechanism of Sb removal by SRB49 was through metal sulfide precipitation with the formation of precipitate Sb_2_S_3_, and no adsorption of Sb was observed on the surface of SRB49. The results demonstrated the roles of pure cultures of SRB species in Sb removal and highlighted the potential environmental importance of Sb tolerant SRB in Sb remediation.

## Data Availability

Not applicable.

## References

[1] He M., Wang N., Long X., Zhang C., Ma C., Zhong Q., Wang A., Wang Y., Pervaiz A., Shan J. (2019). Antimony speciation in the environment: Recent advances in understanding the biogeochemical processes and ecological effects. J. Environ. Sci. (China).

[2] He M., Wang X., Wu F., Fu Z. (2012). Antimony pollution in China. Sci. Total Environ..

[3] Ren M., Ding S., Fu Z., Yang L., Tang W., Tsang D.C.W., Wang D., Wang Y. (2019). Seasonal antimony pollution caused by high mobility of antimony in sediments: In situ evidence and mechanical interpretation. J. Hazard. Mater..

[4] Nishad P.A., Bhaskarapillai A. (2021). Antimony, a pollutant of emerging concern: A review on industrial sources and remediation technologies. Chemosphere.

[5] Sundar S., Chakravarty J. (2010). Antimony toxicity. Int. J. Environ Res. Public Health.

[6] Yamamura S., Iida C., Kobayashi Y., Watanabe M., Amachi S. (2021). Production of two morphologically different antimony trioxides by a novel antimonate-reducing bacterium, *Geobacter* sp. SVR. J. Hazard. Mater..

[7] Xi Y., Lan S., Li X., Wu Y., Yuan X., Zhang C., Yunguo L., Huang Y., Quan B., Wu S. (2020). Bioremediation of antimony from wastewater by sulfate-reducing bacteria: Effect of the coexisting ferrous ion. Int. Biodeterior. Biodegrad..

[8] Zhu Y., Wu M., Gao N., Chu W., An N., Wang Q., Wang S. (2018). Removal of antimonate from wastewater by dissimilatory bacterial reduction: Role of the coexisting sulfate. J. Hazard. Mater..

[9] Wang H., Chen F., Mu S., Zhang D., Pan X., Lee D.-J., Chang J.-S. (2013). Removal of antimony (Sb (V)) from Sb mine drainage: Biological sulfate reduction and sulfide oxidation–precipitation. Bioresour. Technol..

[10] Kulp T.R., Miller L.G., Braiotta F., Webb S.M., Kocar B.D., Blum J.S., Oremland R.S. (2014). Microbiological Reduction of Sb(V) in Anoxic Freshwater Sediments. Environ. Sci. Technol..

[11] Li Y.C., Xu Z., Wu J.X., Mo P. (2020). Efficiency and mechanisms of antimony removal from wastewater using mixed cultures of iron-oxidizing bacteria and sulfate-reducing bacteria based on scrap iron. Sep. Purif. Technol..

[12] Wang L., Ye L., Yu Y., Jing C. (2018). Antimony Redox Biotransformation in the Subsurface: Effect of Indigenous Sb(V) Respiring Microbiota. Environ. Sci. Technol..

[13] Zhang G., Ouyang X., Li H., Fu Z., Chen J. (2016). Bioremoval of antimony from contaminated waters by a mixed batch culture of sulfate-reducing bacteria. Int. Biodeterior. Biodegrad..

[14] Xiao E.Z., Krumins V., Tang S., Xiao T.F., Ning Z.P., Lan X.L., Sun W.M. (2016). Correlating microbial community profiles with geochemical conditions in a watershed heavily contaminated by an antimony tailing pond. Environ. Pollut..

[15] Baek Y.-W., An Y.-J. (2011). Microbial toxicity of metal oxide nanoparticles (CuO, NiO, ZnO, and Sb_2_O_3_) to *Escherichia coli*, *Bacillus subtilis*, and *Streptococcus aureus*. Sci. Total Environ..

[16] Ye L., Chen H., Jing C. (2019). Sulfate-Reducing Bacteria Mobilize Adsorbed Antimonate by Thioantimonate Formation. Environ. Sci. Technol. Lett..

[17] Liu F., Zhang G., Liu S., Fu Z., Chen J., Ma C. (2018). Bioremoval of arsenic and antimony from wastewater by a mixed culture of sulfate-reducing bacteria using lactate and ethanol as carbon sources. Int. Biodeterior. Biodegrad..

[18] Abin C.A., Hollibaugh J.T. (2017). *Desulfuribacillus stibiiarsenatis* sp. nov., an obligately anaerobic, dissimilatory antimonate- and arsenate-reducing bacterium isolated from anoxic sediments, and emended description of the genus *Desulfuribacillus*. Int. J. Syst. Evol. Microbiol..

[19] Lai C.Y., Wen L.L., Zhang Y., Luo S.S., Wang Q.Y., Luo Y.H., Chen R., Yang X.E., Rittmann B.E., Zhao H.P. (2016). Autotrophic antimonate bio-reduction using hydrogen as the electron donor. Water Res..

[20] Muyzer G., Stams A.J.M. (2008). The ecology and biotechnology of sulphate-reducing bacteria. Nat. Rev. Microbiol..

[21] Li X., Xiao H., Zhang W., Li Y., Tang X., Duan J., Yang Z., Wang J., Guan F., Ding G. (2019). Analysis of cultivable aerobic bacterial community composition and screening for facultative sulfate-reducing bacteria in marine corrosive steel. J. Oceanol. Limnol..

[22] Chen W., Xiang F., Fu J., Wang Q., Wang W., Zeng Q., Yu L. (2009). Identification and Phylogenetic Analysis of New Sulfate-Reducing Bacteria Isolated from Oilfield Samples. Z. Nat. C.

[23] Mogensen G.L., Kjeldsen K.U., Ingvorsen K. (2005). *Desulfovibrio aerotolerans* sp nov., an oxygen tolerant sulphatereducing bacterium isolated from activated sludge. Anaerobe.

[24] Okkenhaug G., Zhu Y.-G., He J., Li X., Luo L., Mulder J. (2012). Antimony (Sb) and Arsenic (As) in Sb Mining Impacted Paddy Soil from Xikuangshan, China: Differences in Mechanisms Controlling Soil Sequestration and Uptake in Rice. Environ. Sci. Technol..

[25] Ňancucheo I., Rowe O.F., Hedrich S., Johnson D.B. (2016). Solid and liquid media for isolating and cultivating acidophilic and acid-tolerant sulfate-reducing bacteria. FEMS Microbiol. Lett..

[26] Geets J., Borrernans B., Diels L., Springael D., Vangronsveld J., van der Lelie D., Vanbroekhoven K. (2006). *DsrB* gene-based DGGE for community and diversity surveys of sulfate-reducing bacteria. J. Microbiol. Methods.

[27] Wagner M., Roger A.J., Flax J.L., Brusseau G.A., Stahl D.A. (1998). Phylogeny of dissimilatory sulfite reductases supports an early origin of sulfate respiration. J. Bacteriol..

[28] Zhang J., Wu S.S., Zhao L.H., Ma Q.L., Li X., Ni M.Y., Zhou T., Zhu H.L. (2018). Culture-dependent and-independent analysis of bacterial community structure in Jiangshui, a traditional Chinese fermented vegetable food. LWT-Food Sci. Technol..

[29] Althobiti R.A., Beauchemin D. (2021). Pragmatic method based on on-line leaching and inductively coupled plasma mass spectrometry for risk assessment of the impact of short-term pollution. J. Anal. At. Spectrom..

[30] Kolmert A., Wikstrom P., Hallberg K.B. (2000). A fast and simple turbidimetric method for the determination of sulfate in sulfate-reducing bacterial cultures. J. Microbiol. Methods.

[31] Castillo U., Myers S., Browne L., Strobel G., Hess W.M., Hanks J., Reay D. (2005). Scanning electron microscopy of some endophytic streptomycetes in Snakevine—Kennedia nigricans. Scanning.

